# Safety and Efficacy of Antiviral Drugs and Vaccines in Pregnant Women: Insights from Physiologically Based Pharmacokinetic Modeling and Integration of Viral Infection Dynamics

**DOI:** 10.3390/vaccines12070782

**Published:** 2024-07-16

**Authors:** Bárbara Costa, Maria João Gouveia, Nuno Vale

**Affiliations:** 1 PerMed Research Group, Center for Health Technology and Services Research (CINTESIS), 4200-450 Porto, Portugal; b.c.211297@gmail.com; 2 CINTESIS@RISE, Faculty of Medicine, University of Porto, 4200-319 Porto, Portugal; 3 Department of Community Medicine, Health Information and Decision (MEDCIDS), Faculty of Medicine, University of Porto, 4200-319 Porto, Portugal; 4 Centre for Parasite Biology and Immunology, Department of Infectious Diseases, National Health Institute Dr. Ricardo Jorge, 4000-055 Porto, Portugal; mariajoaogouveia@gmail.com; 5 Center for the Study in Animal Science (CECA/ICETA), University of Porto, 4051-401 Porto, Portugal

**Keywords:** antivirals, pregnancy, human immunodeficiency virus (HIV), hepatitis B virus (HBV), hepatitis C virus (HCV), influenza, cytomegalovirus (CMV), SARS-CoV-2 (COVID-19), PBPK modeling, machine learning, causal inference

## Abstract

Addressing the complexities of managing viral infections during pregnancy is essential for informed medical decision-making. This comprehensive review delves into the management of key viral infections impacting pregnant women, namely Human Immunodeficiency Virus (HIV), Hepatitis B Virus/Hepatitis C Virus (HBV/HCV), Influenza, Cytomegalovirus (CMV), and SARS-CoV-2 (COVID-19). We evaluate the safety and efficacy profiles of antiviral treatments for each infection, while also exploring innovative avenues such as gene vaccines and their potential in mitigating viral threats during pregnancy. Additionally, the review examines strategies to overcome challenges, encompassing prophylactic and therapeutic vaccine research, regulatory considerations, and safety protocols. Utilizing advanced methodologies, including PBPK modeling, machine learning, artificial intelligence, and causal inference, we can amplify our comprehension and decision-making capabilities in this intricate domain. This narrative review aims to shed light on diverse approaches and ongoing advancements, this review aims to foster progress in antiviral therapy for pregnant women, improving maternal and fetal health outcomes.

## 1. Medication Use in Pregnancy: Navegating Challenges, Considerations, Strategies, and the Pursuit of Optimal Health Outcomes

The necessity for medication during pregnancy often arises to manage underlying health conditions. Pregnant women typically require one to three drugs in addition to the recommended intake of iron and vitamins. These drugs serve diverse purposes, ranging from addressing temporary discomforts like nausea or infections of the respiratory or urinary tract to managing chronic conditions such as psychiatric disorders, HIV infection, epilepsy, and rheumatological issues [1,2]. Moreover, medication may become necessary to address conditions that arise during pregnancy, such as high blood pressure, preterm labor, and gestational diabetes [3]. Between 1997 and 2018, there was a notable 35% increase in the proportion of women reporting the use of at least one prescription medication during the first trimester [1,4]. In certain instances, refraining from or discontinuing medication use during pregnancy may pose greater risks than continuing it. Simultaneously, the use of specific medications during pregnancy can elevate the likelihood of birth defects, pregnancy complications, premature birth, infant mortality, or developmental disorders. The impact of medication on both the pregnant woman and the fetus can be influenced by the dosage, timing of administration during pregnancy, existing health conditions, and concurrent medication usage [5,6]; however, this has not been correctly measured by the scientific community.

**Figure 1:**
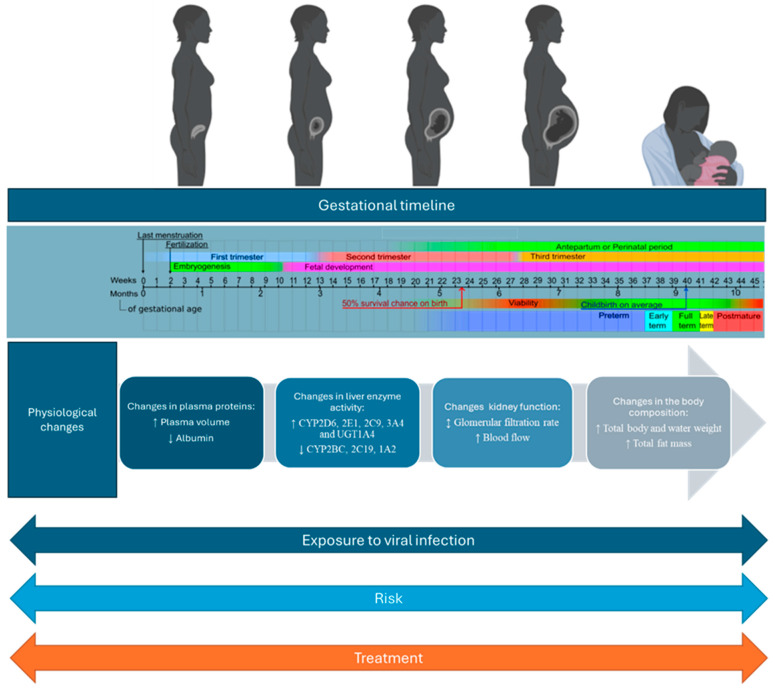
Gestational timeline and physiological changes in pregnancy: implications for pharmacokinetics, cytochrome P450 activity, and viral infection treatment.

This knowledge gap persists due to the exclusion of pregnant individuals from studies assessing the safety of new medications [7,8,9]. The scarcity of sufficient data on dosing during pregnancy is a prevalent issue among the majority of approved drugs [10], despite the use of the Food and Drug Administration’s (FDA) categorization system which ranges from Category A (safe) to Category X (contraindicated) [11] according to their teratogenic risk, which poses challenges for informed decision-making. Historically, pregnant women have been systematically excluded from clinical trials, resulting in a significant lack of specific data on how drugs affect this population, including information on dosing, efficacy, and safety [12]. This issue is compounded by the substantial changes pregnancy brings to drug pharmacokinetics, impacting absorption, distribution, metabolism, and excretion, necessitating tailored studies to establish appropriate dosages [13], considering how heightened plasma volume, altered drug-binding plasma proteins, increased cardiac output, and shifts in body composition affect drug metabolism and distribution [14]; see Figure 1. Regulatory constraints and liability concern further contribute to the scarcity of data as pharmaceutical companies may hesitate to conduct extensive research, fearing regulatory obstacles and legal consequences [15]. This leaves healthcare providers facing the complex challenge of weighing the potential risks of medication use during pregnancy against the benefits of treating maternal conditions.

Addressing these challenges necessitates a concerted effort to conduct extensive research and gather comprehensive data on drug safety in pregnant individuals, aiming for optimal maternal and fetal health outcomes [16,17]. This effort involves enhancing collaboration among stakeholders, including research institutions, governmental agencies such as the CDC, pharmaceutical companies, healthcare providers, and patient advocacy groups. Allocating funding and resources to establish dedicated research centers or networks focused on studying medication use during pregnancy can facilitate large-scale studies, data collection, and analysis [3]. Additionally, expanding pregnancy registries can improve data collection on medication safety through increased participation and standardized methods. Strengthening adverse event reporting systems, like the FDA MedWatch Program [1], or creating Physiologically Based Pharmacokinetic Model-Informed Frameworks can enable researchers to strategically focus on medications with the greatest potential impact on maternal and fetal health, maximizing the utilization of limited resources (e.g., M-CERSI) [18,19,20]. Monitoring plasma drug concentrations and considering how pregnancy influences medication pharmacokinetics and pharmacodynamics, including biologics, is essential. Biospecimens such as amniotic fluid, umbilical cord blood, placental tissue, meconium, umbilical cord tissue, and newborn hair provide valuable insights into fetal drug exposure across different therapies [21].

On the other hand, viral infections during pregnancy can further perturb the maternal PK/PD system, leading to altered drug responses and potentially impacting both the mother and the developing fetus. The maternal immune system’s response to viral infections also influences pregnancy outcomes and the effectiveness of treatments. Comprehensive care for pregnant women with chronic diseases involves addressing postpartum adjustments, such as modifying treatment regimens for breastfeeding and considering changes during breastfeeding. The inadequate management of chronic conditions during pregnancy can lead to preterm birth, low birth weight, and congenital anomalies. Managing viral infections discovered during pregnancy also requires specialized considerations. Pregnant women with HIV face unique challenges in balancing maternal health and fetal well-being, selecting appropriate treatments, and closely monitoring therapy adjustments throughout pregnancy [22,23,24]. To effectively treat pregnant women with viral infections, clinicians must carefully consider the interplay between pregnancy-induced changes and the effects of the viral condition on the maternal PK/PD system. Therefore, it is critical to thoroughly study the safety and effectiveness of antiviral medications in pregnant women to maximize treatment success and ensure the health of both mother and child. Pregnancy increases susceptibility to infections and the potential for dormant infections to reactivate, heightening the risks associated with untreated infections [25,26]. In cases of severe systemic infection, additional changes in drug absorption, distribution, metabolism, and excretion may occur during pregnancy. Some antiviral drugs are designed solely for maternal treatment, while others, such as antiretrovirals, must prevent vertical transmission while ensuring fetal safety [27,28]. It is crucial to rigorously assess pharmacokinetic data and dosing regimens to determine if therapeutic levels are achieved during pregnancy or if adjustments are necessary. Key considerations include study objectives, design, target concentrations, the adequacy of pharmacokinetic sampling schedules, the appropriateness of pharmacometrics analysis, and data transparency.

This narrative review aims to explore medication use during pregnancy to manage viral infections by synthesizing the existing literature. Here, we identify gaps and strategies to optimize health outcomes for both mothers and their unborn children, considering various viral infections and their risks throughout gestation.

## 2. Viral Infections Relevant to Pregnancy

Viral infections relevant to pregnancy encompass a variety of pathogens, including viruses, bacteria, protozoa, and fungi. These infections, contracted before or during pregnancy, can be transmitted to the fetus through various routes, including congenitally during gestation, perinatally during labor and childbirth, and postnatally through breastfeeding [26,29]. Specifically, this review will delve into viruses posing significant risks during pregnancy, as well as those for which optimal treatment strategies are still underexplored. Such viruses include human immunodeficiency virus (HIV), hepatitis B and C virus (HBV and HCV), cytomegalovirus (CMV), influenza A virus (IAV), and the recently emerged SARS-CoV-2.

### 2.1. Human Immunodeficiency Virus (HIV) in Pregnancy

HIV belongs to the Lentivirus genus and Retroviridae family, characterized by single-stranded, positive-sense, enveloped RNA [30]. It targets CD4+ lymphocytes, integrating into the host–cell genome, and leads to acquired immunodeficiency syndrome (AIDS) [31]. HIV-1 is globally prevalent and more virulent, while HIV-2 is confined to West Africa. Transmission occurs through blood, semen, and vaginal fluids, with mother-to-child transmission (MTCT) possible during pregnancy, delivery, and breastfeeding [32]. Maternal HIV-1 infection correlates with adverse pregnancy outcomes such as premature labor and miscarriage. Vertical transmission routes include intrauterine, intrapartum, and postpartum transmission, influenced by maternal viral load, immune status, and birth mode [26]. In women with HIV infection, additional infections affecting the placenta, fetal membranes, genital tract, and breast tissue, as well as systemic infections in both the mother and the infant, have been demonstrated to elevate the risk of MTCT of HIV [33].

Maternal HIV diagnosis relies on virologic assays, particularly polymerase chain reaction (PCR) tests, which are considered the gold standard for detecting HIV infection in both infants and adults. For infant HIV diagnosis, virologic assays are essential due to maternal immunoglobulin IgG transfer, with PCR assays serving as the gold standard. Prompt testing within days of birth and subsequent follow-ups are crucial [34]. All pregnant women should undergo HIV screening, with immediate testing recommended for those with unknown HIV status during labor or delivery. Point-of-care testing for infants can enhance early diagnosis, especially in resource-limited settings [35]. Clinical management strategies, including cesarean section and antiretroviral therapy, significantly reduce the risk of transmission. During pregnancy, the placenta’s antiviral response limits vertical transmission, although HIV persists in peripheral blood monocytes despite antiretroviral therapy [36,37]. Placental alterations, including inflammation and vascular malperfusion, contribute to adverse outcomes. Placental macrophages and T regulatory cells play roles in controlling MTCT [38]; therefore, Highly Active Antiretroviral Therapy (HAART) is vital, but the timing and type of HAART initiation influence maternal and fetal outcomes. HAART may increase preterm delivery risk due to potential toxicity and immune dysregulation [39]. Assessing antiretroviral safety in pregnancy is crucial for optimizing treatment. The prevention of MTCT is a significant achievement with HAART, recommended for all pregnant women with HIV regardless of CD4+ count. Elective cesarean delivery and neonatal prophylaxis reduce transmission risk, with zidovudine (ZDV) being a common prophylactic treatment [40]. ZDV/lamivudine (3TC) and ZDV are more effective in reducing the risk of mother-to-child transmission, with ZDV/3TC also showing a reduced risk of stillbirth [41]. However, concerns remain regarding toxicity in HIV-exposed uninfected infants, emphasizing the need for monitoring and follow-up [22].

### 2.2. Hepatitis B and C Virus (HBV and HCV) in Pregnancy

HBV and HCV are hepatotropic viruses that belong to the Hepadnaviridae family; they are bloodborne pathogens posing significant risks during pregnancy, primarily through MTCT [42]. HBV, a globally prevalent pathogen, is transmitted mainly in the third trimester, with transmission rates reaching up to 90% [43,44]. Despite effective strategies like immunoprophylaxis and antiviral treatments, vertical transmission rates remain high due to uneven vaccine coverage and prophylaxis failures [45]. HBV surface antigen (HBsAg) is a crucial viral marker, indicating hepatitis B virus infection, while HCV-RNA signifies active hepatitis C virus infection. During pregnancy, women are routinely screened for HBsAg, followed by further testing for HBV-DNA and serologic markers. Likewise, infants born to HCV-positive mothers undergo testing for HCV-RNA and are closely monitored for up to 18 months post-birth. It is advised to minimize invasive prenatal procedures, and cesarean section is not recommended solely for preventing the vertical transmission of hepatitis viruses. However, perinatal HBV transmission can be effectively prevented by identifying HBV-positive pregnant women (HBsAg-positive) and promptly administering the hepatitis B vaccine and immune globulin to newborns within 12 h of delivery [46]. Initiating antiviral therapy for HBV at 28–32 weeks’ gestation can reduce transmission risk for high viral loads [47]. HBV transmission mechanisms include transplacental leakage, placental infection, and the crossing of infected maternal blood cells into the placenta. Maternal immune changes during pregnancy may promote HBV transmission, leading to immune-tolerant HBV infection in the fetus [48]. Conversely, HCV infection alters placental morphology, increasing the risk of complications such as preterm birth and stillbirth [49]. The risk of HCV transmission is heightened when there is a high maternal serum viral load during delivery, indicating active viremia [50]. This risk escalates proportionately with increasing levels of viral load above 105 IU/mL and peaks at levels exceeding 107 IU/mL [51,52]. Tenofovir (TDF) first-line antiviral medication is advised for such instances, beginning at week 28 of pregnancy and continuing until birth. Three months after giving birth, treatment may continue. Every pregnant patient with an HBV diagnosis needs to be referred to a physician who specializes in treating HBV infections for follow-up care. Because liver health and HBV infection can fluctuate over time, it is imperative to have regular monitoring throughout life. Additionally, elevated maternal serum ALT levels in the 12 months preceding pregnancy and/or during delivery are indicative of a higher viral replication rate, potentially leading to more extensive hepatic damage and subsequent ALT elevation [53,54]. Early screening of HCV-exposed infants is essential for prompt treatment and prevention of complications, emphasizing the importance of comprehensive management strategies to mitigate vertical transmission risks of HBV and HCV during pregnancy [22].

### 2.3. Influenza in Pregnancy

Influenza viruses are RNA viruses from the family Orthomyxoviridae. Influenza viruses, particularly type A, cause respiratory symptoms and spread mainly through airborne droplets. Pregnant women face increased susceptibility and risks of severe complications from influenza, especially in later gestational stages [55]. Influenza virus infection during pregnancy poses significant risks to both the mother and fetus, leading to various acute and chronic complications. A Centers for Disease Control and Prevention (CDC) study published in October 2020 found that flu infection during pregnancy is associated with an increased risk of pregnancy loss, a reduction in the birthweight of full-term newborns, and an increased risk of late pregnancy loss (defined as pregnancy loss after 13 weeks gestation). Pregnant women with respiratory illness symptoms and fever were also found to have an increased risk of preterm birth. The study included 11,277 pregnant women from India, Peru, and Thailand during the 2017 and 2018 flu seasons. Only 13% of study participants had been vaccinated against flu. This study underscores the potential importance of flu vaccination in pregnant women to prevent poor pregnancy outcomes associated with flu infection [56].

Although the vertical transmission of the influenza virus to the fetus is rare, maternal infection can still affect fetal health through mechanisms like placental damage, apoptosis, and viral replication, potentially leading to adverse outcomes such as intrauterine growth restriction and birth defects [57]. Pregnancy may increase susceptibility to infection and raise the chance of major illness outcomes due to physiological and immunological changes [58]. Pregnant women exhibit reduced interferon responses, heightening their vulnerability to severe outcomes [59]. The virus can cause placental damage, disrupting nutrient and oxygen exchange between the mother and fetus [60]. Dysregulated maternal immune responses, including excessive inflammation and cytokine production, can contribute to adverse pregnancy outcomes and fetal damage [61]. Offspring born to mothers who experienced influenza virus infection during pregnancy face an increased risk of long-term neurological disorders like schizophrenia [62]. In chronic complications, influenza infection during pregnancy can trigger long-term cardiovascular issues due to vascular dysfunction and inflammation [63]. Pregnant women infected with influenza, including types like swine flu (H1N1), may experience more severe symptoms and complications compared to non-pregnant individuals, often resulting in higher hospitalization rates and mortality. Respiratory complications such as acute respiratory distress syndrome (ARDS) and secondary bacterial or viral pneumonia can occur, contributing to maternal morbidity and mortality [64].

Preventive measures for pregnant individuals include receiving the influenza vaccine, practicing good hygiene, and seeking prompt medical attention if symptoms develop [65]. Understanding these mechanisms is crucial for effectively managing and mitigating the risks associated with influenza virus infection during pregnancy [22]. Further research is imperative to gain a deeper understanding of these outcomes and to differentiate influenza from other pathogens. This differentiation is particularly important given that influenza viruses, while posing risks to pregnancy outcomes, do not fit into the traditional TORCH group. There is a need for more mechanistic studies, to enhance our understanding of influenza’s unique impact on maternal and fetal health.

### 2.4. Cytomegalovirus (CMV) in Pregnancy

The CMV, a DNA virus belonging to the Betaherpesvirinae subfamily of the Herpesviridae family [66], is associated with severe clinical outcomes in cases of congenital infection, particularly prevalent among individuals with limited socioeconomic resources [67]. The risk of primary CMV infection during pregnancy is notable because a relatively large proportion of women of reproductive age are CMV-seronegative [68]. Unlike other infectious diseases, CMV presents an increased risk of fetal involvement during pregnancy due to the high prevalence of seropositivity among women of childbearing age [69]. The transmission of CMV during pregnancy can range from 20 to 70% during primary maternal infections, with a reduced risk during recurrent infections [70]. Placental dysfunction is a critical factor in the development of congenital CMV infection [71]. CMV replication in cytotrophoblasts leads to placental edema, fibrosis, and compromised nutrient and oxygen transport to the fetus [72]. CMV-induced placental damage results from a combination of molecular mechanisms, such as impaired extracellular matrix development, the IL-10-mediated inhibition of matrix metalloproteinases, and the activation of the peroxisome proliferator-activated receptor [72]. Untreated CMV infections pose significant risks of adverse pregnancy outcomes, developmental disabilities, and long-term health complications for the newborn, including developmental delays and neurodevelopmental disorders. Neonates infected with CMV may display symptoms like intrauterine growth retardation (IUGR), purpura, jaundice, hepatosplenomegaly, microcephaly, hearing impairment, and thrombocytopenia [73]. Long-term complications, such as neurological disorders and sensory impairments, are observed in approximately 40–60% of neonates symptomatic at birth [74].

Diagnosing congenital CMV infection involves serological testing for CMV-specific antibodies (IgM, IgG, and IgG avidity) combined with PCR assays to detect viral DNA in maternal fluids and amniotic fluids [75]. Universal neonatal CMV screening using PCR assays on saliva or urine samples shows promise in identifying high-risk infants, though differentiating congenital from perinatal infection remains challenging [76]. Preventative measures for congenital CMV infection include educating pregnant women on hygiene practices and administering CMV hyperimmune globulins or antiviral drugs in specific cases. However, there is a lack of consensus on screening and preventive strategies which indicates a need for more research to establish evidence-based guidelines. While antiviral medications like valaciclovir, ganciclovir, and valganciclovir demonstrate efficacy in inhibiting CMV replication, there are no officially approved treatments for CMV infection during pregnancy [77]. The existing guidelines recommend that any antenatal therapy for CMV should be provided as part of a research protocol [78], and further research is needed to assess their safety and effectiveness in treating neonatal complications [79]. Studies have revealed significant gaps in knowledge and awareness of CMV among pregnant women and healthcare professionals. This lack of understanding hinders effective prevention and management efforts, highlighting the importance of education and training programs [80]. Evaluations of screening strategies have shown varying cost-effectiveness results, emphasizing the need to identify reliable and sensitive screening tests and establish mechanisms for implementation and monitoring [81]. CMV vaccine development continues to be a major public health priority, as highlighted by the absence of an available active vaccine. Research in this area is crucial to prevent congenital CMV infections and their associated complications [76,82].

### 2.5. Severe Acute Respiratory Syndrome Coronavirus 2 (SARS-CoV-2) in Pregnancy

Lastly, severe acute respiratory syndrome coronavirus 2 (SARS-CoV-2), the virus responsible for coronavirus disease 2019 (COVID-19), is famously known for the global pandemic. Belonging to the Coronaviridae family [83], it is a newly discovered β-Coronavirus with a positive-sense single-stranded RNA virus. SARS-CoV-2 primarily spreads through droplets and aerosols during close contact, with an incubation period of 2 to 14 days [84]. Neonatal and pediatric cases of SARS-CoV-2 are often mild and linked to family clusters, with evidence suggesting minimal vertical transmission during maternal infection [85,86]. However, diagnosing congenital infection remains challenging, with only a small percentage of neonatal cases confirmed as congenital infections [87]. The association between maternal SARS-CoV-2 infection, placental histomorphology, and perinatal outcomes remains uncertain, with limited published studies on how SARS-CoV-2 affects placental structure in infected pregnant women. However, recent research aimed to investigate these effects by conducting a retrospective cohort study on 47 pregnant women with confirmed SARS-CoV-2 infection, matched with non-infected controls. The study found that while only one of the infected cases showed SARS-CoV-2 immunoreactivity in the syncytiotrophoblasts, there were significant histomorphological differences in placentas from SARS-CoV-2-infected pregnancies compared to the control group. These differences included higher rates of decidual vasculopathy, maternal vascular thrombosis, and chronic histiocytic intervillositis in the placentas from SARS-CoV-2-infected pregnancies. Furthermore, active SARS-CoV-2 infection during pregnancy was associated with a lower gestational age at delivery, a higher rate of cesarean section, lower fetal-placental weight ratio, and poorer Apgar scores. Notably, active, symptomatic, and severe-critical maternal SARS-CoV-2 infection, along with placental inflammation, were linked to an increased risk of preterm delivery. Additionally, altered placental villous maturation and severe-critical maternal SARS-CoV-2 infection were associated with an elevated risk of poor Apgar scores at birth and maternal mortality, respectively [88]. In contrast, a prospective cohort study on 30 pregnant women infected with SARS-CoV-2 and their neonates found that maternal anti-SARS-CoV-2 Spike antibodies could cross the placenta during pregnancy, resulting in neonates acquiring antibodies at birth. However, all neonates tested negative for SARS-CoV-2 infection, and the immunohistochemical staining for Spike protein in placental tissues was negative. The study also indicated a correlation between maternal and neonatal levels of total anti-SARS-CoV-2 Spike antibodies, with higher concentrations observed in pregnant women with moderate to severe/critical disease [89].

The severity of maternal SARS-CoV-2 infection was associated with ischemic placental pathology, potentially leading to adverse pregnancy outcomes. Despite this, placental tissues did not show detectable SARS-CoV-2 infection, suggesting that placental infection is rare. Instead, SARS-CoV-2 infection during pregnancy primarily induces unique inflammatory responses at the maternal–fetal interface, involving maternal T cells and fetal stromal cells. Additionally, maternal–fetal immune responses to SARS-CoV-2 did not compromise the T-cell repertoire or initiate IgM responses in neonates. Overall, these findings provide insights into the maternal–fetal immune responses triggered by SARS-CoV-2 and highlight the rarity of placental infection during maternal viral infection [90].

Pregnant women with COVID-19 are at an increased risk of developing severe complications, requiring hospitalization, and facing adverse effects on pregnancy outcomes, emphasizing the importance of timely treatment and preventive measures [91]. Therefore, preventing SARS-CoV-2 spread is crucial, with efforts focusing on vaccination, surveillance, and tracking new variants. Limited data on vaccine safety during pregnancy and breastfeeding are available, but initial reports suggest maternal vaccination with mRNA-based vaccines may confer passive immunity to neonates [92]. Continued monitoring is necessary to assess outcomes in vaccinated pregnant women and their infants.

Extensive research on the causes of viral infections during pregnancy is vital in protecting both mothers and babies from emerging pandemics. There is a need to advance our knowledge of antiviral treatments, vaccines, and their effects during pregnancy so we can enhance our ability to manage viral infections in pregnant women more effectively, reducing risks and improving outcomes for both mothers and babies (while mitigating the impact of future pandemics and epidemics). The aforementioned highlights the importance of ongoing research to refine antiviral therapies for pregnant individuals, enhancing our ability to respond effectively to future public health crises.

## 3. Overview of Antiviral Treatment in Pregnant Women: Safety and Efficacy Considerations

Antiviral treatment during pregnancy is complex, balancing infection management with maternal and fetal safety. In this section of the narrative review, the goal is to explore antiviral medications in pregnancy, addressing risks, management, and therapeutic efficacy, since untreated viral infections can harm both mother and fetus, emphasizing the need for effective interventions [93]. We will also examine the safety and effectiveness of these treatments in pregnancy, emphasizing evidence-based approaches to enhance maternal and fetal health outcomes [13,23,58]. While these medications hold promise in mitigating the impact of viral illnesses, their utilization necessitates a nuanced understanding of their potential benefits and risks in the unique physiological setting of pregnancy.

### 3.1. Treatment and Management of HIV

The recommendations for the use of antiretroviral drugs during pregnancy emphasize the importance of selecting appropriate initial regimens for individuals who are starting ART for the first-time during pregnancy and have no evidence of resistance to regimen components. For those already on suppressive ART regimens when pregnancy occurs, it is recommended to continue with those regimens, unless there are concerns about safety or efficacy during pregnancy. Changes to ART regimens should ideally be timed to achieve viral suppression before attempting pregnancy. The recommendations provide guidance on selecting regimens, emphasizing shared decision-making between patients and providers based on the benefits of ART and the potential risks to pregnant individuals and their fetuses [94]. The recommendations are detailed in tables categorized by drug class and recommendation category, with no preference indicated for one agent or regimen over another within each category. Overall, the WHO recommends ART for all pregnant women living with HIV, regardless of their CD4 count or clinical stage. ART should be started as early as 14 weeks of gestation or as soon as possible during pregnancy. The goal is to achieve and maintain HIV viral suppression to undetectable levels and to reduce the risk of mother-to-child transmission of HIV [94]. The most common ART regimens recommended for pregnant women with HIV are tenofovir disoproxil fumarate (TDF) + emtricitabine (FTC) + efavirenz (EFV) or raltegravir (RAL) and TDF + FTC + lopinavir/ritonavir (LPV/r). Pregnant women starting ART should be monitored monthly or bimonthly, depending on adherence and the length of virological suppression. Monitoring visits should be as close as possible to the predicted delivery date [41,95]. HIV viral load should be tested every two months during pregnancy, including for up to 36 weeks of gestation [96]. Regular testing helps assess treatment efficacy and ensures viral suppression. Decisions regarding ART adjustments or switches should be individualized. Factors considered include the person’s treatment history, adherence, tolerability, and potential risks associated with ART exposure during pregnancy. If switching is due to insufficient safety and efficacy data during pregnancy, this should be discussed with the pregnant woman, considering her preferences [97,98]. ART should be initiated immediately for women whose follow-up starts late in the second or third trimester. Integrase strand transfer inhibitors (INSTIs) like raltegravir (RAL) or dolutegravir (DTG) can be used for rapid viral load decline. The risk of neural tube defects associated with DTG has been reduced, and its efficacy is well-established. If HIV viral load is not undetectable in the third trimester, it is necessary to test for resistance and consider changing to or adding INSTIs if not already in this class to achieve rapid viral load decline [99]. For women with a HIV viral load >400 copies/mL at weeks 34–36, it is necessary to plan elective cesarean section at week 38 [100]. Zidovudine (ZDV) prophylaxis during labor and delivery is essential [101]. While these regimens are generally safe, ongoing studies are needed to further evaluate their safety and efficacy in pregnant women. The context of the SARS-CoV-2 pandemic has also impacted HIV services, emphasizing the importance of maintaining proper care.

Ensuring the safety and efficacy of HIV treatment regimens in pregnant women is paramount for maternal and fetal health. Recent research highlights key considerations regarding various drug regimens. DTG-containing regimens stand out for their superior virologic efficacy in suppressing HIV during pregnancy. When combined with emtricitabine and tenofovir alafenamide fumarate (TAF), DTG-containing regimens have been associated with the lowest risk for maternal and fetal adverse events [102]. Additionally, studies suggest that DTG-containing regimens outperform efavirenz (EFV)-based regimens in HIV suppression. Emtricitabine/Tenofovir Alafenamide Fumarate (FTC/TAF) is considered safe during pregnancy and contributes to effective viral suppression. Combining FTC/TAF with DTG may further reduce adverse pregnancy outcomes [103]. A significant concern arises regarding recommendations for ART, which often lag behind guidelines for pregnant adults living with HIV. This lag results in pregnant individuals receiving older, less desirable ART regimens, posing a considerable concern. This issue arises from insufficient data to recommend the use of newer drugs like doravirine, intramuscular rilpivirine, bictegravir, cabotegravir, and ibalizumab during pregnancy, posing challenges in determining the most appropriate and effective treatment regimens for pregnant women with HIV. As a result, healthcare providers often resort to using older, more established ART regimens to ensure the well-being of both the mother and the fetus, despite the potential limitations of these regimens.

Untreated maternal HIV infection can lead to an increased risk of adverse pregnancy outcomes, but some anti-HIV medications have been linked to health problems in pregnancy, including weight gain, diabetes, depression, and adverse effects on maternal health [104]. Despite the optimization of maternal health through ART, adverse pregnancy outcomes among HIV-positive women remain worse than those among HIV-negative women, indicating a persistent discrepancy in outcomes [105]. Neonates exposed to maternal ART may experience adverse effects such as anemia, thrombocytopenia, liver function abnormalities, preterm birth, low birth weight, and congenital malformations. Fetal exposure to antiretroviral drugs during pregnancy can result in hematological, hepatic, and mitochondrial changes, as well as an increased risk of preterm birth, low birth weight, neonatal mortality, fetal growth restriction, congenital malformations, and viral resistance. The transference of maternal antiretroviral drugs to the fetus through the placenta can lead to potential adverse effects on fetal development and health [25,105]. Until more robust data become available, healthcare providers must rely on existing guidelines and evidence-based practices to make informed decisions regarding ART regimens for pregnant women living with HIV, prioritizing the health of both the mother and the child. However, we can do better.

### 3.2. Treatment and Management HBV and HCV

In the case of HBV, the US Centers for Disease Control and Prevention (CDC) recommends that the HBV vaccine birth dose should be given within 12 h to neonates born to women with HBV infection. Antiviral prophylaxis is recommended if the maternal HBV DNA is >200,000 IU/mL, as a conservative choice to minimize vertical HBV transmission. First-line antiviral therapy with tenofovir (TDF) is recommended, commencing at week 28 of pregnancy and continuing until delivery. This regimen may extend for up to three months postpartum. By reducing the viral load, antiviral therapy significantly decreases the risk of transmitting the virus to the newborn [106]. The ideal timing of antiviral initiation and cessation in pregnancy is not yet well defined. Antiviral therapies with efficacy and safety include nucleotide/nucleoside analogue polymerase inhibitors: tenofovir disoproxil fumarate (TDF), lamivudine, and telbivudine [107,108,109].

HBV treatment involves a different class of medications, including nucleos(t)ide analogs (NAs) and interferons. These medications inhibit HBV replication by targeting the viral polymerase. TDF and entecavir are commonly used NAs. Interferon-based therapies stimulate the immune system to fight HBV. However, they are less commonly used due to side effects [110,111]. Regular monitoring throughout pregnancy is paramount to ensure timely adjustments to the treatment plan as needed and enables healthcare providers to closely track the disease. Ongoing research aims to address these gaps and improve outcomes for pregnant women with HBV. Additionally, it is necessary to explore treatment response disparities based on how HBV genotypes can further refine treatment approaches, optimizing outcomes for both mother and child. Research should explore differences in treatment response based on HBV genotypes and investigate the role of combination therapy (e.g., NAs plus interferon) during pregnancy. Long-term follow-up studies are essential to assess outcomes for both mothers and infants.

Direct-acting antiviral (DAA) therapy is now recommended for nearly all patients with chronic HCV infection, directly targeting the virus to inhibit its replication, leading to high cure rates with minimal side effects. However, there are few clinical trials evaluating the safety and efficacy of DAAs in pregnancy, their use during pregnancy is not approved [112,113]. Antiviral therapy is recommended before considering pregnancy. Research on HCV treatment during pregnancy faces several gaps, including limited safety and efficacy data, a lack of prospective studies, insufficient understanding of patient preferences, limited research on maternal linkage to care strategies, and the need for a pregnancy exposure registry to collect real-world data. The TiP-HepC Registry, which stands for Treatment in Pregnancy for Hepatitis C, is an important initiative aimed at addressing the knowledge gap regarding the safety of DAA medications during pregnancy. Its primary objective is to gather real-world data on HCV treatment outcomes specifically among pregnant individuals. By providing healthcare providers with vital insights into treatment safety, efficacy, and long-term outcomes, the registry facilitates informed decision-making and enhances patient care. Key objectives of the registry include collecting data on pregnant individuals undergoing HCV treatment, monitoring treatment response and maternal-infant health outcomes, and informing future treatment guidelines. Through the voluntary contributions of healthcare providers, the registry compiles comprehensive information, including patient demographics, treatment regimens, laboratory results, and follow-up data. The registry supports evidence-based practice, aids ongoing research efforts, and improves care delivery for pregnant individuals with HCV. In essence, the TiP-HepC Registry represents a significant stride forward in our understanding and management of HCV during pregnancy [114]. Women of reproductive age with HCV should be counselled about the benefit of antiviral treatment prior to pregnancy to improve health and eliminate the risk of vertical transmission. Women who become pregnant while on DAA therapy (with or without ribavirin) need to discuss the risks versus benefits of continuing treatment with their physicians [94].

### 3.3. Treatment and Management of Influenza

The treatment of influenza in pregnant women involves the use of antiviral agents, such as oseltamivir, which have been shown to be safe and effective in reducing the severity and duration of symptoms [115]. Antiviral treatment should not be delayed while awaiting respiratory infection test results, and a patient’s vaccination status should not affect the decision to treat.

### 3.4. Treatment and Management of CMV

Currently, there is no specific treatment for CMV infection during pregnancy. Ganciclovir and valganciclovir are antiviral medications used to treat CMV infections, but when considering their use in pregnant women, the safety and efficacy profiles must be carefully evaluated [78]. Ganciclovir has been shown to be effective in treating CMV infections in pregnant women. However, it is associated with a risk of teratogenicity, meaning it can cause birth defects if taken during pregnancy. Ganciclovir has also been linked to bone marrow suppression, which can lead to a decrease in blood cell counts [116], its use in pregnant women is not recommended. Valganciclovir, the prodrug of ganciclovir, has a similar efficacy profile but may have a more favorable safety profile for use in pregnant women. Valganciclovir has been shown to have a lower risk of teratogenicity compared to ganciclovir, and it may be better tolerated in terms of bone marrow suppression [117]. However, the data on the use of valganciclovir in pregnant women are more limited, and its safety and efficacy in this population are still being evaluated [118].

Valacyclovir is classified as Pregnancy Category B, indicating no evidence of risk in humans based on animal studies. Studies have not shown an increased risk of birth defects with the use of acyclovir (a related drug) during pregnancy, which is converted to valaciclovir in the body. While generally considered safe, common side effects of valaciclovir include nausea, headache, and abdominal pain. Pregnant women using valaciclovir should be monitored closely for any adverse effects on both the mother and the fetus. Valaciclovir is effective in treating CMV infections by inhibiting viral replication, reducing symptoms, and potentially preventing transmission to the fetus [119,120]. Early treatment with antivirals like valaciclovir may reduce the risk of congenital CMV infection in newborns. Initiating treatment promptly upon diagnosis is crucial to maximize the efficacy of valaciclovir in managing CMV infections during pregnancy. While valaciclovir is generally considered safe and effective for treating CMV in pregnant women, close monitoring and careful consideration of the risks and benefits are essential. Consultation with a healthcare provider is crucial to make informed decisions regarding the use of valaciclovir during pregnancy.

For 20 years, hyperimmune globulin (HIG) has been used as an immunomodulatory treatment for CMV infections in pregnant women [121,122,123,124]. Observational studies have reported an excellent safety and efficacy profile for preventing congenital CMV disease using HIG at a dosage of 200 units/kg [79]. A study analyzed factors predictive of infant outcomes in pregnant women with primary CMV infection. Among 157 women treated with an average of 2 doses of high-dose HIG, several factors were identified: maternal viremia, which predicted fetal infection and neonatal outcome; HIG administration was associated with the resolution of viral DNAemia; and abnormal ultrasounds predicted both symptoms at birth and long-term sequelae [125]. While observational studies support the use of HIG, conclusive evidence from randomized prospective studies is still lacking. Current recommendations do not routinely advocate for immunoglobulin treatment in pregnant women with primary CMV infection. Many pregnant women with primary CMV infection have asymptomatic or mild courses, and the majority of infants born to CMV-infected mothers do not develop severe complications. Some experts advocate for supportive care and close monitoring rather than routine treatment, as the long-term impact of HIG on the health of both mother and baby remains unclear [126]. Balancing short-term prevention with potential long-term effects is challenging. Despite advancements in the management of CMV infections in pregnant women, several research gaps persist. There is a need to find effective and safe treatment regimens for CMV in pregnant women, considering factors such as gestational age, viral load, maternal immunity, and potential adverse effects on the fetus. Research is needed to assess the long-term outcomes of infants exposed to antiviral medications or other treatments for CMV during pregnancy, including neurodevelopmental outcomes and potential risks of drug exposure [127]. Essentially, further research is needed to determine the optimal timing and duration of treatment for CMV in pregnant women to maximize efficacy while minimizing potential harm. It is imperative to conduct comparative studies to assess the efficacy and safety of various treatment options for pregnant women with CMV infection [128]. These studies should encompass antiviral medications, hyperimmune globulin, and supportive care to provide comprehensive insights for clinical decision-making. Targeted research is needed to stratify the risk among pregnant women with CMV infection, enabling the identification of those at heightened risk of adverse outcomes. Addressing these research gaps is crucial to enhance the management of CMV infections in pregnant women, improve maternal and fetal outcomes, and guide evidence-based clinical practice in this complex area of obstetrics and infectious diseases [129].

### 3.5. Treatment and Management of SARS-CoV-2

The management of SARS-CoV-2 in pregnant women has been an area of active research and clinical practice. While the landscape of available antivirals is evolving, the following are some of the key antivirals currently used to treat pregnant women infected with SARS-CoV-2. Remdesivir has been authorized for the treatment of SARS-CoV-2 in certain clinical settings [130]. It is used safely and effectively in pregnant women with COVID-19, with no significant safety concerns reported, often being considered a first-line antiviral option for pregnant women with moderate to severe SARS-CoV-2 infections [131]. Another oral antiviral medication that is authorized for the treatment of SARS-CoV-2 in certain high-risk populations is nirmatrelvir/ritonavir. Limited data are available on the use of this combination in pregnant women, and its safety and efficacy in this population are still in evaluation. Caution and consultation with a healthcare provider is recommended when considering the use of nirmatrelvir/ritonavir in pregnant women, [132].

Research is needed to evaluate the long-term safety and efficacy of specific antiviral medications, such as remdesivir, in pregnant women with SARS-CoV-2, considering potential maternal and fetal outcomes. Studies are required to determine the most effective treatment strategies for SARS-CoV-2 in pregnant women, including the timing, duration, and combination of antiviral medications, to improve outcomes while minimizing risks. Further research is needed to evaluate the impact of SARS-CoV-2 infection and its treatment on maternal health, pregnancy outcomes, and fetal development, including potential long-term effects. Investigation into immunomodulatory therapies like corticosteroids or monoclonal antibodies in pregnant women with SARS-CoV-2 aims to manage inflammation and prevent severe disease while ensuring maternal and fetal safety [133,134]. Understanding the pharmacokinetics and pharmacodynamics of antiviral medications in pregnancy, including drug metabolism, placental transfer, and interactions with physiological changes, is essential. Ethical considerations regarding the inclusion of pregnant women in clinical trials of treatments are crucial, covering informed consent, protection of maternal and fetal rights, and equitable access to novel therapies. Addressing these concerns is vital to optimize the management of viral infections during pregnancy, enhance maternal and fetal outcomes, and guide evidence-based clinical practice in this vulnerable population [15].

Recommended dosing regimens for HIV, HBV/HCV, influenza, CMV, and SARS-CoV-2 in pregnant women involve a combination of antiretroviral therapy, antiviral prophylaxis, antiviral agents, and vaccines, balancing safety and efficacy considerations (Table 1). However, there remains a critical need for further research into the safety and efficacy of these regimens specifically tailored to pregnant women. This is especially pertinent in the context of the COVID-19 pandemic, which has disrupted HIV services and heightened the risk of HIV transmission [135]. Ensuring access to appropriate treatment and care for pregnant women is essential in mitigating the risks of maternal and fetal morbidity and mortality associated with these infections.

### 3.6. Importance of Careful Dosage Determination and Adherence

Ensuring accurate dosage determination and adherence to antiviral treatment during pregnancy is paramount for achieving optimal outcomes for both the mother and the developing fetus [136,137]. A proper dosage is crucial for achieving the desired therapeutic effects of antiviral medications. Underdosing may render the treatment ineffective, leading to persistent viral activity and potential harm to the mother and the fetus. Conversely, overdosing can result in adverse effects, posing risks to maternal health and fetal development. Pregnancy induces physiological changes that can significantly impact drug metabolism and pharmacokinetics, leading to under or overdosing [138]. These changes can cause fluctuations in drug levels in the body, highlighting the need for close monitoring and personalized dosing adjustments for each pregnant individual. By considering these variations, healthcare providers can optimize treatment efficacy while minimizing potential risks associated with inadequate drug levels. So, consistent adherence to prescribed treatment regimens is essential for managing viral infections during pregnancy effectively. Missing doses or inconsistent adherence can compromise the efficacy of antiviral therapy, allowing viral replication to persist and potentially leading to the development of drug-resistant viral strains.

## 4. Vaccines and Pregnant Population: Emerging Areas and Strategies

Vaccination in pregnant populations is a critical area of focus to protect both mothers and infants from viral infections. The COVID-19 pandemic has highlighted the importance of vaccination during pregnancy. Pregnant individuals are at an increased risk of severe disease if they contract SARS-CoV-2 [139]. Fortunately, observational data have shown that the benefits of SARS-CoV-2 vaccination outweigh the potential risks for pregnant, postpartum, and lactating women. The World Health Organization, Centers for Disease Control and Prevention, and professional organizations recommend SARS-CoV-2 vaccination for this population [140].

Recent studies monitoring pregnant individuals who received SARS-CoV-2 vaccines have not raised any specific safety concerns related to pregnancy. Although pregnant women were initially excluded from clinical trials of SARS-CoV-2 vaccines, observational data have rapidly accumulated, confirming that the benefits of vaccination outweigh the potential risks [140,141]. Regarding the other viral infections discussed here, there is currently no specific HIV and HCV vaccine recommended for pregnant women. In the case of influenza and HBV, vaccines are generally safe during pregnancy. HBV can prevent vertical transmission to the newborn [142]. Inactivated influenza vaccines are recommended for all pregnant women to prevent maternal influenza infection and reduce complications during pregnancy [143]. For CMV, there is no specific vaccine available, pregnant individuals should follow hygiene measures to reduce exposure to CMV [144].

Prophylactic vaccines are generally the most recommended for viral infections during pregnancy, offering direct protection to the mother and indirect protection to the fetus [145]. Therapeutic vaccines, specifically gene vaccines, may have roles in select cases but require thorough evaluation considering the physiological and immunological changes in pregnancy [146]. In Table 2, we summarize information regarding prophylactic and therapeutic vaccines for viral infections during pregnancy. The suitability of different types of vaccination (prophylactic and therapeutic) varies depending on the viral infection and pregnancy stage. Prophylactic vaccines, such as those for influenza and SARS-CoV-2, are commonly used to prevent viral infections during pregnancy [147]. Therapeutic and gene vaccines are still in the early development stages for viruses like HIV, HBV/HCV, CMV, and SARS-CoV-2. Factors influencing vaccine suitability include the virus’s nature, pregnancy stage, and availability of safe candidates. It is crucial to consider specific viral infections, pregnancy stages, and potential risks for both mother and fetus when selecting vaccine types. Some infections may not recommend certain vaccine types, like live-attenuated vaccines, due to safety concerns.

HIV-ongoing research related to prophylactic and therapeutic vaccines during pregnancy include the multi-stage HIV vaccine regimen. Researchers from the George Washington University Vaccine Research Unit, in collaboration with other institutions, have developed a multi-stage HIV vaccine regimen. The first stage of this vaccine strategy aims to produce broadly neutralizing antibodies (bnAbs) capable of targeting a wide range of HIV variants. The vaccine showed favorable safety profiles and induced the targeted immune response in 97% of vaccinated individual [148]. Additionally, the National Institute of Allergy and Infectious Diseases (NIAID) has launched a Phase 1 clinical trial evaluating three experimental HIV vaccines based on a messenger RNA (mRNA). This technology, similar to that used in SARS-CoV-2 mRNA vaccines, holds promise for developing preventive HIV vaccines [149].

In the context of HBV and HCV co-infection in HIV-infected individuals, ongoing research on vaccines aims to enhance the understanding of mechanisms that promote HBV infection in this population. Strategies are being explored to reduce the prevalence of HBV co-infection among individuals living with HIV [150]. It is necessary to understand the specific mechanisms that contribute to increased susceptibility to HBV infection in individuals with HIV. Factors such as immune suppression, altered immune responses, and shared routes of transmission between HIV, HBV, and HCV may play a role in promoting HBV infection in HIV-infected individuals. Vaccines that can effectively prevent HBV infection in individuals living with HIV aim to enhance immune responses, provide long-lasting protection, and reduce the risk of HBV co-infection in the HIV-infected population [151]. Strategies to reduce the prevalence of HBV co-infection in HIV-infected individuals include targeted vaccination programs, early screening for HBV, and integrated care models that address both HIV and HBV management. Efforts are being made to improve access to vaccination, promote adherence to vaccination schedules, and enhance awareness about the importance of HBV prevention in the context of HIV care. By reducing the burden of HBV co-infection in individuals with HIV, these research efforts have the potential to improve health outcomes, reduce liver-related complications, and enhance the overall well-being of HIV-infected individuals. Strategies aimed at preventing HBV co-infection can have a significant impact on public health by reducing the transmission of HBV, improving treatment outcomes, and lowering the overall disease burden in the HIV-infected population.

Influenza vaccines, though not pregnancy-specific, are continually evolving through ongoing research. Recommended for all pregnant women, they prevent maternal influenza infection and related complications during pregnancy. Current research aims to enhance vaccine efficacy, safety, and immune responses, adapting to the dynamic influenza virus [143]. Efforts focus on expanding vaccine coverage, especially among high-risk groups like older women and those with pre-existing conditions. Additionally, research addresses vaccination disparities among socioeconomically disadvantaged women. Safety assessments of influenza vaccination during pregnancy evaluate potential risks of adverse birth outcomes and maternal non-obstetric adverse events. The World Health Organization advocates influenza vaccination for all pregnant women, leading many countries to implement vaccination programs, though coverage varies.

An effective CMV vaccine holds promise for preventing the majority of birth defects associated with congenital CMV infections. Candidate vaccines, including live-attenuated, protein subunit, DNA, and viral-vectored approaches, are under clinical evaluation. Subunit approaches target key CMV proteins, such as pp65, IE1, and glycoprotein B (gB), which induce cytotoxic T cells and neutralizing antibodies [152]. However, recent insights into CMV entry pathways highlight opportunities for improvement. Notably, a 5-subunit pentameric complex is crucial for viral entry into endothelial and epithelial cells, suggesting a potential target for vaccine enhancement [153]. Antibodies may inhibit post-entry CMV spread between cells, limiting viral replication and dissemination to the fetus [154,155]. Next-generation vaccine candidates, including peptides, recombinant proteins, DNA, viral vectors, and inactivated CMV, are in preclinical development, offering hope for a successful candidate [154,156].

SARS-CoV-2 vaccines, including mRNA-based vaccines, have been authorized for use during pregnancy [157]. Ongoing studies monitor safety and effectiveness in pregnant populations. The mRNA vaccines like those used for SARS-CoV-2 offer innovative approaches to immunization [158]. In pregnant populations, gene vaccines can provide robust immune responses without the use of live viruses, enhancing safety profiles for both mother and fetus. However, the implications of gene vaccines in pregnancy require careful consideration due to limited data on long-term effects and potential interactions with maternal and fetal immune systems [159,160]. They offer enhanced safety profiles by eliciting robust immune responses without live viruses, minimizing risks to both mother and fetus. Their rapid development timelines are advantageous for swiftly mutating viruses or public health crises. The adaptable nature of gene vaccine platforms allows for tailored formulations, addressing the unique physiological and immunological changes of pregnancy. Gene vaccines show potential in improving protection against viral infections for both pregnant women and their fetuses. However, adapting regulatory frameworks is crucial to ensure these vaccines are safely approved, monitored, and surveilled post-market. Continued research is essential to fully understand their safety, effectiveness, and optimal use in pregnant populations. Challenges include ensuring safety, addressing limited clinical evidence, and understanding how these vaccines interact immunologically and in terms of vertical transmission. Collaboration among researchers, developers, and regulators is key to advancing safe and effective gene vaccines for pregnant women, thereby enhancing maternal and fetal health protection against viral infections [161].

Recent advancements in maternal vaccination have led to the authorization of the RSV (Respiratory Syncytial Virus) vaccine for pregnant women in both the USA and the EU. In August 2023, the US Food and Drug Administration (FDA) and in September 2023, the European Medicines Agency (EMA) approved the vaccine based on robust clinical data demonstrating its ability to significantly reduce severe RSV infections in infants during their first six months of life. This approval marks a crucial step in preventing RSV-related complications in newborns, as the virus commonly causes conditions like bronchiolitis and pneumonia [162]. Clinical trials have established the safety and efficacy of Abrysvo, the Pfizer-developed RSV vaccine, when administered to pregnant women. By triggering an immune response in the mother, the vaccine transfers protective antibodies to the fetus through the placenta, offering passive immunity to newborns during their early vulnerable months. Abrysvo is recommended for pregnant women between 32–36 weeks gestation, providing infants with protection from birth up to 6 months old. Research indicates that maternal vaccination with Abrysvo can reduce severe RSV illness in infants by 91% [162]. Common side effects reported in pregnant women receiving the vaccine include pain at the injection site, headache, muscle pain, and nausea. While clinical trials showed a slightly higher rate of preterm births in the vaccine group compared to placebo, this difference was not statistically significant [163]. The introduction of the RSV vaccine for pregnant women is expected to have a significant public health impact by decreasing RSV-related hospitalizations and medical visits, thereby improving neonatal outcomes and reducing the strain on healthcare systems during peak RSV seasons. This authorization represents a pivotal advancement in maternal and neonatal health. Continued surveillance and research will be crucial to monitor the long-term benefits and potential risks associated with maternal RSV vaccination.

Vaccines play a vital role in protecting pregnant populations from viral infections. Excluding pregnant women from vaccine safety and efficacy trials poses risks, including the lack of evidence-based guidance, disparities in care and outcomes, missed opportunities for data collection, ethical concerns, and implications for healthcare workers. Addressing these risks is crucial to ensure equitable access to potentially life-saving interventions during public health emergencies [164]. By addressing challenges through tailored vaccine development, robust regulatory oversight, safety monitoring, and epidemiological insights, we can optimize vaccine strategies to safeguard the health of pregnant women and their infants.

## 5. Understanding the Pharmacokinetics and Pharmacodynamics of Antiviral Medications and Vaccines in Pregnant Women

**Figure 2:**
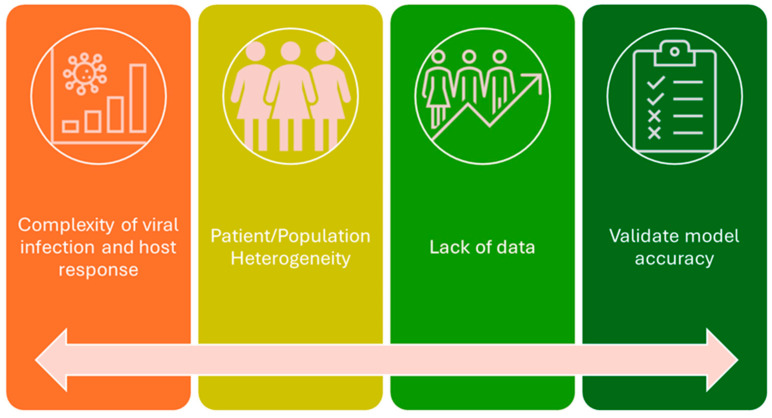
Current challenges of modeling viral infection drug treatment in pregnant women.

Pregnancy initiates a complex interplay of physiological changes that profoundly influence the interactions among antiviral drugs, vaccines, and the maternal–fetal unit [14,138]. The presence of viral infections further complicates this environment, amplifying the challenges of managing medications and vaccines during gestation [165]. These challenges encompass alterations in gastrointestinal function impacting drug absorption and shifts in immune responses affecting vaccine efficacy. Understanding these intricacies is crucial for optimizing maternal and fetal health outcomes, guiding clinical decision-making, and tailoring treatment strategies for this unique population (Figure 2). To address these complexities, innovative methodologies such as in vitro placenta perfusion studies and Physiologically Based Pharmacokinetic (PBPK) modeling play pivotal roles. Placenta perfusion studies provide valuable insights into the passage of medications and vaccines across the placental barrier, illuminating potential fetal exposure and safety concerns [166]. PBPK modeling facilitates the simulation of maternal–fetal drug dynamics, considering the physiological changes inherent in pregnancy. These approaches offer a comprehensive framework for evaluating the safety and efficacy of interventions during gestation [167]. Overcoming the challenge of limited data can be achieved through advanced techniques such as machine learning and causal inference. Machine learning algorithms can analyze vast datasets to identify patterns and predict outcomes, thereby enhancing our understanding of the effects of antiviral drugs and vaccines on pregnant women [168]. Causal inference methodologies enable us to infer causal relationships from observational data, providing valuable insights into the impacts of interventions on maternal and fetal health. By leveraging these innovative approaches, researchers and healthcare providers can overcome data limitations, enhance our understanding of pregnancy-related pharmacology, and ultimately improve outcomes for pregnant women and their babies.

### 5.1. Leveraging In Vitro Studies

In recent years, there has been a heightened recognition of the complexities surrounding viral infections during pregnancy, leading to an increased reliance on pharmaceutical interventions. This shift has been paralleled by significant advancements in our understanding of placental drug transfer and metabolism. Investigating how drugs traverse the placenta is crucial for comprehending their metabolic pathways and ensuring their safe administration during gestation [169,170]. This section aims to explore diverse techniques developed for evaluating the transplacental transfer of drugs and xenobiotics, providing insights into their effectiveness and safety in managing viral infections during pregnancy.

The placenta acts as a barrier between the maternal and fetal circulations, but many drugs can still cross it, potentially affecting the fetus. Some drugs are intentionally administered to the mother to treat fetal conditions, while others may cause harm. Drug transfer across the placenta depends on factors like molecular weight, lipid solubility, and protein binding. Three types of drug transfer are recognized: complete transfer (type 1), exceeding transfer (type 2), and incomplete transfer (type 3). The mechanisms of transfer include simple diffusion, facilitated diffusion, and active transport, each with different requirements and implications for drug movement. Active transport, for instance, involves carrier-mediated transfer against a concentration gradient and requires energy in the form of ATP. Various drug transporters play roles in these processes, with their expression and distribution changing throughout gestation [171]. Various in vitro models, including primary trophoblastic cells, immortal cell lines, and placental tissue explants, have been developed to study drug transport across the placenta [172]. These models offer insights into transporter protein functions and xenobiotic metabolism. Ex vivo human placental perfusion models provide a more representative approach to studying drug transport, allowing for the assessment of drug transporters, metabolism, and tissue binding.

A recent study exemplifies this crucial area of research by investigating the impact of infections at the maternal–fetal interface on pregnancy outcomes and fetal health. Researchers explored how these cells contribute to antiviral defenses and immune signaling during pregnancy using organoids derived from human placental trophoblasts and decidua. By examining the differential responses of trophoblast and decidua organoids to viral infections, the study shed light on the intricate mechanisms involved in protecting the fetus from pathogens. Moreover, the development of a co-culture model demonstrated how trophoblast-derived factors can protect decidua cells from infection, highlighting the complex interplay between maternal and fetal tissues in maintaining a healthy pregnancy. Overall, this research not only advances our understanding of congenital infections but also underscores the importance of studying drugs in pregnancy to ensure their safety and effectiveness for both maternal and fetal health [173]. Nucleoside reverse transcriptase inhibitors (NRTIs) like zidovudine and lamivudine have relatively high placental transfer, while protease inhibitors like nelfinavir and ritonavir have lower placental transfer [27]. Plus, the expression and activity of drug transporters like P-glycoprotein and organic anion transporters in the placenta can influence the extent of fetal exposure to these antiviral drugs [174].

Several studies investigated the transplacental transfer and tissue accumulation of the HIV integrase inhibitor dolutegravir using ex vivo human cotyledon perfusion models. In one study, dolutegravir concentrations were measured in both maternal and fetal compartments after 3 h of perfusion. The results showed that dolutegravir crossed the placenta significantly, with fetal exposure being considerable and accumulation in placental tissue being notable. The fetal-to-maternal ratio was around 34%, indicating substantial transfer to the fetus [175]. Another study found a fetal-to-maternal concentration ratio of 0.6, indicating moderate to high transfer across the placenta. These findings suggest potential clinical implications for dolutegravir in preventing the mother-to-child transmission of HIV and highlight the need for further investigation into potential risks for the fetus [176]. A study involving lamivudine, an antiviral drug used for HBV, conducted using HepG2 cells, a hepatoma cell line, demonstrated the effectiveness of lamivudine in inhibiting HBV replication in vitro. This in vitro model not only facilitated the screening of antiviral drugs but also enabled the investigation of the pharmacokinetics (pk) and pharmacodynamics (pd) of lamivudine in pregnant women. By utilizing this model, researchers were able to assess the efficacy and safety of lamivudine specifically in the context of treating HBV in pregnant women, providing valuable insights into the drug’s potential benefits and risks in this population [177,178].

Cerveny et al. investigated the placental transport of the nucleoside analog entecavir (ETV), a drug commonly used to treat chronic hepatitis B. Despite its efficacy, ETV is not recommended for use in pregnant women due to insufficient data on its safety during pregnancy. The study evaluates the contribution of various nucleoside and efflux transporters to the placental kinetics of ETV using in vitro, ex vivo, and in situ methods. Nonetheless, the key findings are that equilibrative nucleoside transporters (ENTs), particularly ENT1, significantly contribute to the placental uptake of ETV. Other transporters such as concentrative nucleoside transporters (CNTs), P-glycoprotein (ABCB1), breast cancer resistance protein (ABCG2), and multidrug resistance-associated protein 2 (ABCC2) do not play a significant role in the placental kinetics of ETV. Inhibition studies with nucleosides and NBMPR, a nucleoside transport inhibitor, support the involvement of nucleoside transporters in ETV uptake. The findings suggest that ETV, despite its hydrophilic nature, can cross the placental barrier mainly through ENT-mediated transport. Overall, these results provide valuable insights into the mechanisms underlying the placental transport and may contribute to a better understanding of its safety profile in pregnant women, potentially informing clinical decisions regarding ETV therapy during pregnancy [179].

Research on influenza has demonstrated that oseltamivir is capable of crossing the placenta, as both oseltamivir and its active metabolite (oseltamivir carboxylate) have been detected in cord blood. Moreover, these substances reach higher concentrations in cord blood compared to what has been observed in ex vivo placental models previously. Several specific studies have contributed significantly to this field. Huang et al. investigated oseltamivir’s transplacental transfer using ex vivo placental models, shedding light on its dynamics [180]. Ehrenstein et al. have also contributed to our understanding of oseltamivir’s safety during pregnancy [181]. Regarding SARS-CoV-2, limited data are available on the placental transfer of antiviral drugs used to treat the disease, such as remdesivir and molnupiravir. However, studies are ongoing to assess the fetal exposure and safety of these medications in pregnant women. Continued research using in vitro and ex vivo placental models will be crucial in evaluating the transplacental pharmacokinetics of emerging COVID-19 therapies. Nonetheless, Vero E6 cells (African green monkey kidney cells) were used to propagate SARS-CoV-2 (the virus causing COVID-19). This model allowed researchers to study viral replication and test antiviral drugs [182].

Understanding placental drug transfer and metabolism is critical for ensuring medication safety during pregnancy, particularly in managing viral infections. Future research directions include refining in vitro and ex vivo models to better mimic placental physiology and integrating in silico techniques with experimental data. These advancements enable the development of safer and more effective medications for use during pregnancy, thereby ultimately enhancing maternal and fetal health outcomes. In silico techniques such as computational models and simulations serve as complementary tools to validate experimental data.

### 5.2. PK/PBPK Modeling of Antiviral Drugs in Pregnancy

**Figure 3:**
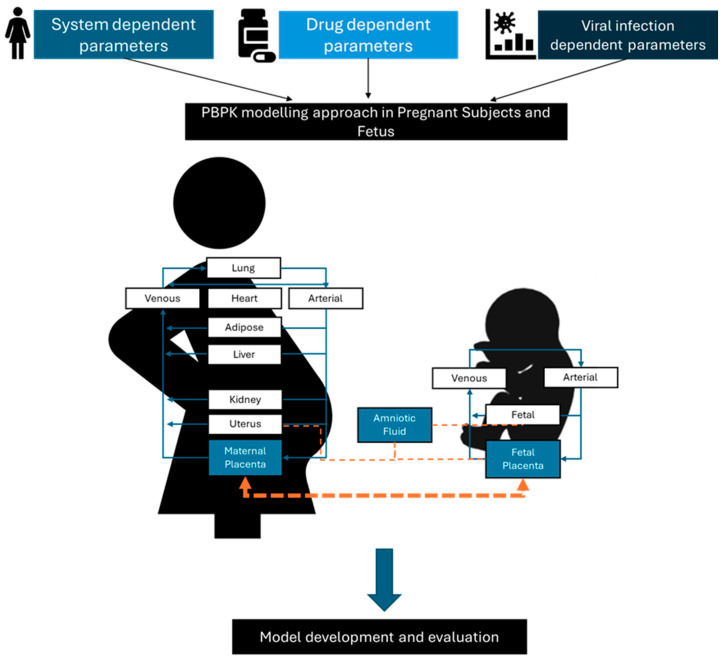
PBPPK modeling and integration of viral infection model into PK/PBPK approach.

PBPK modeling serves as a valuable computational tool capable of predicting drug disposition during pregnancy by integrating drug-specific properties with time-varying physiological changes. This approach enables the study of inter-individual variabilities and their impact on drug disposition, offering insights beyond what is observed in traditional studies. PBPK models also incorporate gestational age-related physiological variations (Figure 3), enabling the prediction of gestational age-dependent pharmacokinetics for various drugs [183]. This enhances our understanding of antiviral drug efficacy during pregnancy and facilitates model-informed drug development (MIDD) [184]. These models can anticipate clinical pharmacokinetic outcomes affected by both extrinsic and intrinsic factors in diverse populations. They aid in predicting formulation- and food-dependent drug pharmacokinetics, informing clinical study design prior to first-in-human (FIH) trials. Additionally, PBPK models can predict and quantify drug–drug interactions (DDIs) mediated by drug-metabolizing enzymes or transporters, guiding dose selection and criteria for inclusion/exclusion in clinical trials [185,186]. However, despite its significant contributions, PBPK modeling has limitations in assessing drug safety and efficacy during pregnancy. Challenges arise from the large number of parameters required and the limited availability of in vivo data to verify individual parameters, leading to the potential confounding of model predictions. Regulatory support, usability for clinicians, and validation challenges hinder the widespread adoption of PBPK modeling in clinical practice [187].

PBPK models have been developed to predict the maternal and fetal PK of ARVs like dolutegravir and raltegravir during pregnancy. Physiological changes during pregnancy, such as increased blood flow and volume, were also incorporated to reliably predict maternal PK profiles in the second and third trimesters [188]. The models also can predict fetal exposure, with the predicted umbilical vein concentrations aligning well with in vivo data. PopPK modeling can be applied to optimize the dosing of ARVs like efavirenz, dolutegravir, and rilpivirine in pregnant women [189]. For example, a popPK model for efavirenz predicted lower exposure in the third trimester, suggesting the potential need for a dose increase to maintain therapeutic levels [190]. Additionally, PBPK models have been extended to assess ARV distribution into lymphoid tissues and fetal plasma during pregnancy [189]. This provides insights into potential viral reservoirs and fetal exposure, which are important for evaluating the risk of mother-to-child transmission.

PBPK models can incorporate genetic polymorphisms that affect drug metabolism and transport, which can help identify subpopulations that may require dose adjustments and be used to determine the optimal timing of ARV dosing during pregnancy to maximize efficacy and minimize fetal exposure [191]. The case of efavirenz presents an intriguing example of how genetic polymorphisms in drug-metabolizing enzymes can significantly influence pharmacokinetics and clinical outcomes because individual responses to efavirenz can vary considerably due to genetic factors, particularly in pregnant women [192]. Metabolically, efavirenz undergoes extensive hepatic metabolism primarily via the cytochrome P450 (CYP) enzyme system, with significant contributions from enzymes such as CYP2B6 and CYP3A4. Additionally, several UDP-glucuronosyltransferases (UGTs) participate in the glucuronidation of efavirenz. Genetic polymorphisms in these enzymes can exert a profound impact on efavirenz pharmacokinetics. Of particular interest are polymorphisms in the CYP2B6 gene, which can lead to significant variability in enzyme activity. Common variants such as CYP2B6*6 (516G→T) and CYP2B6*18 (983T→C) have been associated with altered efavirenz metabolism [193,194]. Individuals carrying these slow-metabolizer genotypes may experience higher plasma efavirenz concentrations, potentially leading to adverse effects or improved virologic response. The clinical implications of these genetic variations are multifaceted. Pregnant women with slow-metabolizer genotypes may exhibit elevated plasma efavirenz concentrations, predisposing them to central nervous system (CNS) side effects such as vivid dreams and dizziness. Interestingly, the impact of these polymorphisms can vary by ethnicity, with potential differences in CNS events and virologic outcomes [195]. In response to these considerations, clinicians may need to adjust efavirenz dosing based on genotype to optimize therapeutic outcomes.

Scott et al. assessed the dosing of tenofovir disoproxil fumarate (TDF)/emtricitabine (FTC) for HIV pre-exposure prophylaxis (PrEP) during pregnancy through clinical trial simulation. They used popPK modeling and simulation to evaluate the adequacy of standard dosing in maintaining protective drug levels during pregnancy. The simulation indicated that standard dosing resulted in a considerable proportion of individuals having suboptimal drug levels during pregnancy, particularly in the third trimester. However, doubling the standard dose during pregnancy showed improvement but still fell short of ensuring protective drug levels for all individuals. This simulation underscores the necessity for prospective studies on pregnancy-adjusted dosing for TDF/FTC PrEP [196,197].

Another example is the study by De Sousa Mendes et al. aimed to assess the impact of pregnancy on the PK of three renally excreted antiretroviral drugs: tenofovir (TFV), emtricitabine (FTC) and lamivudine (3TC). They developed whole-body PBPK models and utilized time-varying pregnancy-related physiological parameters to simulate PK profiles [198]. The models successfully predicted the disposition of these drugs in both non-pregnant and pregnant populations. The study found that renal clearance, which follows changes in glomerular filtration, increased during pregnancy, with maximum clearance increases of approximately 30% for TFV, FTC, and 3TC. Furthermore, the study concluded that pregnancy PBPK models are valuable tools for quantifying drug exposure changes during pregnancy for renally excreted drugs. These models can aid in evaluating alternative dosing regimens to optimize drug therapy during pregnancy. The study also addressed the challenges of conducting pharmacokinetic studies in pregnant women by providing an alternative approach to predicting changes in drug disposition. This is particularly important for drugs like TFV, where limited data on pharmacokinetics during pregnancy are available [198,199]. In the case of population pharmacokinetic studies, it has been shown that the pharmacokinetics of oseltamivir, a drug used in the treatment of influenza, are altered in pregnant women. Specifically, the systemic exposure of its active metabolite, oseltamivir carboxylate, is reduced by approximately 30% [200]. This suggests that the currently recommended doses of oseltamivir may need to be increased in pregnant women to achieve comparable exposure with non-pregnant women.

Regarding CMV infections, PBPK models were developed for valganciclovir and acyclovir, with the latter also evaluating their performance in predicting maternal PK at different stages of pregnancy and concentrations in the umbilical vein at delivery [201]. Abduljalil et al. further expanded this work by predicting the maternal and fetal drug exposure of acyclovir and emtricitabine during pregnancy and at delivery using a PBPK modeling approach [202]. Sychterz et al. reviewed the application of PBPK models in pregnant populations, highlighting the need for further expansion in areas such as drug–drug interactions and non-CYP enzymes [203]. In the case of COVID-19, PK/PBPK modeling has been used to translate non-pregnant adult PBPK models for remdesivir (RDV) and its metabolites to pregnant women. The developed PBPK model captured RDV and its metabolites’ throughout pregnancy. Specifically, the predictions of AUC and C max for RDV and its metabolites fell within a twofold error range, with approximately 60% of predictions achieving accuracy within a 10% error margin [204,205]. Moreover, defining the CYP and UGTs involved in RDV’s metabolism is essential for creating a good model. RDV is primarily metabolized by CYP3A4, CYP2C8, and CYP2D6, and UGT1A1, UGT1A3, UGT1A9, UGT2B4, UGT2B7, and UGT2B15. Understanding the involvement of these enzymes and their polymorphisms is crucial for predicting the drug’s metabolism and elimination in pregnant women [206]. Polymorphisms in CYP and UGT enzymes can significantly affect the pharmacokinetics of drugs, and different alleles can result in varying enzyme activity, leading to differences in drug metabolism and elimination. Similarly, UGT enzymes also exhibit polymorphisms, which can impact the glucuronidation of drugs.

Moreover, the PBPK modeling, widely used in drug development, is being explored for its potential to support the development of oral vaccines with alpha-tocopherol as an adjuvant. Tegenge et al. developed a PBPK model for emulsified alpha-tocopherol in adjuvanted influenza vaccines, predicting its rapid clearance from the injection site and subsequent accumulation in draining lymph nodes, with slow systemic absorption and eventual storage in adipose tissue. This model sheds light on the biodistribution and fate of alpha-tocopherol in vaccine adjuvants, offering insights into its immunological effects and informing regulatory risk-benefit analyses [207]. Saldanha et al. further contributed to this area by constructing the first PBPK model for an oral vaccine utilizing alpha-tocopherol as an adjuvant. Their model considers factors influencing alpha-tocopherol’s pharmacokinetics, notably its LogP value, which affects its tissue delivery. Based on these findings, an optimal formulation comprising nanoparticles with a 150 mg dose within a 200 mL volume was proposed [208]. Vaccination remains vital for public health, and the integration of PBPK modeling into vaccine development holds promise for enhancing vaccine efficacy [209].

Unlike traditional vaccines, genetic vaccines such as vectorized DNA or mRNA vaccines have two major PK phases: a pharmacological phase involving biodistribution, assimilation, gene translation, and epitope presentation, followed by an immunological phase similar to that of traditional vaccines. Due to this multi-phased mode of action, the clinical outcome of genetic vaccines exhibits more variability than that of traditional vaccines. The increased variability necessitates the regulation of genetic vaccines similarly to biotherapeutics to ensure better efficacy and safety. Naasani et al. propose a structural PK model to predict sources of variability, biodistribution, and dose optimization to maximize the safety and efficacy of genetic vaccines, at three levels: vaccine active material, expressed epitopes, and generated immunity. These studies should be performed before market launch and widespread utilization. Implementing PK studies at these levels enables optimal dose stratification for each subpopulation based on factors such as age, sex, weight, and metabolic activity. The article also highlights the importance of accurate administration methods to avoid adverse effects, such as myocarditis or blood clotting, potentially initiated during the pharmacological phase [210].

In addition to forecasting drug concentration profiles, pharmacokinetic modeling plays a crucial role in assessing drug–drug interactions (DDIs), particularly vital in patients managing multiple conditions. DDIs can affect pharmacokinetics or pharmacodynamics, leading to adverse effects or altered efficacy, especially in cases of polypharmacy or comorbidities. Pharmacokinetic models can serve to evaluate DDIs by simulating the concurrent administration of multiple drugs, identifying potential culprits that may alter metabolism or transport mechanisms, quantifying the impact on drug exposure, and guiding dose adjustments to mitigate adverse effects. For instance, in a pregnant woman managing gestational diabetes, who has mild renal impairment, and requires antenatal supplementation with iron and calcium, a pharmacokinetic assessment of potential DDIs becomes imperative for tailoring dosing regimens to ensure both maternal and fetal safety and efficacy. Furthermore, the limited understanding of the combined impact of DDIs and pregnancy on drug pharmacokinetics highlighted the significance of comprehending the mechanisms, extent, and clinical implications of DDIs in pregnant women and the clinical relevance of these interactions due to physiological changes during pregnancy. Such alterations could potentially compromise treatment efficacy and safety for both the mother and the newborn [211,212].

Although PBPK models are valuable tools for evaluating antiviral drug pharmacokinetics in pregnant individuals, they face several limitations (see Table 3). One challenge is their development based on specific populations, potentially limiting their generalizability across different demographics. Some models lack thorough validation against clinical data, raising uncertainties in their predictive accuracy and risking misestimations of drug exposure and potential adverse outcomes during pregnancy. Another important limitation stems from the incomplete consideration of physiological changes during pregnancy. Current models may not fully incorporate alterations in gastrointestinal absorption or enzyme activity, leading to inaccuracies in drug exposure predictions. Moreover, while some models consider genotype impacts on drug metabolism, they may overlook other genetic factors influencing drug response in pregnancy. All of these hinder their translation into clinical practice [213].

Integrating viral infection models into PBPK models can enhance their predictive capabilities by incorporating disease-specific factors, such as viral load dynamics, immune response modulation, and tissue tropism. These integrated models provide a more comprehensive understanding of drug pharmacokinetics in the context of viral infections during pregnancy. For example, in the case of HIV infection, viral dynamics models can simulate the impact of varying viral loads on drug distribution and elimination rates, allowing for more accurate predictions of antiretroviral drug concentrations in pregnant women. Furthermore, the integration of viral infection models enables the assessment of drug efficacy and resistance development over time. By modeling the interplay between drug pharmacokinetics and viral replication kinetics, researchers can identify optimal dosing regimens to achieve viral suppression while minimizing the risk of drug resistance. This approach also facilitates the evaluation of combination therapy strategies to enhance treatment outcomes and prevent virologic failure during pregnancy.

### 5.3. Integration of Viral Dynamics

The COVID-19 crisis has exposed significant gaps in our understanding of viral kinetics and immune responses within the host, hindering both the discovery of new therapies and the optimization and personalization of existing ones. Mathematical modeling can unravel the complex dynamics of virus–host interactions across various spatial and temporal scales, encompassing processes from viral transport to initial infection sites, the evasion of host defenses, and the infection of target cells. This field, initially shaped by the complexities of understanding the immune response in HIV infection, has paved the way for the development and refinement of these models. Ranging from target-cell-limited models to those integrating adaptive immune responses, mathematical modeling has provided invaluable insights into therapeutic strategies for diverse viral infections (Figure 3).

The integration of viral dynamics and PBPK models is a powerful approach to enhance the evaluation of dose regimens and minimize adverse effects in viral infections. Huang et al. proposed a Bayesian approach for estimating antiviral efficacy in HIV dynamic models [214]. Canini et al. and Zitzmann et al. emphasize the role of viral kinetic modeling in understanding disease progression, optimizing drug regimens, and predicting treatment outcomes for infectious diseases [215,216]. These studies collectively underscore the value of integrating viral dynamics and PBPK models in enhancing the evaluation and management of viral infections. Furthermore, by incorporating viral infection dynamics into PBPK models, researchers can optimize treatment regimens, assess drug exposure, and predict treatment outcomes more effectively.

The Chigutsa et al. study presents a novel approach to support the selection of the optimal dose of bamlanivimab, a monoclonal antibody for treating SARS-CoV-2 caused by SARS-CoV-2. The research integrates PBPK modeling with viral dynamic modeling to predict drug exposure and viral clearance in the body, especially in the lung tissue. The PBPK model estimates the concentration of bamlanivimab needed in the lung tissue to maintain viral neutralization for up to 4 weeks in 90% of patients. Meanwhile, the viral dynamic model predicts the viral clearance based on drug concentration and dose. Through these models, a dose range between 175 and 500 mg of bamlanivimab was suggested to maintain target concentrations in the lung tissue, while a 700 mg dose was predicted to achieve maximum viral elimination. The study emphasizes the importance of open access in silico models for rapid drug development, especially during a pandemic. The approach provides a framework for selecting the most effective dose of bamlanivimab before its first-in-human clinical trial. Additionally, the study highlights the need for further research into the tissue distribution and metabolism of antiviral agents like remdesivir using PBPK models to optimize clinical dosing regimens [217,218].

Modeling viral dynamics and drug interactions during pregnancy poses significant challenges due to physiological changes, limited accurate models, concerns about toxicity and immunogenicity, and data gaps regarding gestational age-related physiological alterations. However, mechanistic computational models hold promise for enhancing our understanding of immune response dynamics, refining treatment strategies, and advancing antiviral therapy development. Mechanistic computational models offer valuable insights into cellular signaling pathways, cytokine crosstalk, and cell–cell communication in immunology. They enable the exploration of complex biological hypotheses involving multiple signals and cell interactions, facilitating a comprehensive understanding of immune responses. Whether simulating signaling pathways, investigating cytokine crosstalk in T cells, or examining cellular behavior at various levels, mechanistic computational models serve as powerful tools for unraveling the intricacies of immunology.

### 5.4. Machine Learning, and Causal Inference Technique

AI and machine learning offer innovative solutions to hurdles encountered in pharmacokinetic modeling. A significant challenge is the limited availability of data, which AI algorithms can address through data augmentation and imputation. By generating synthetic data points or filling in missing values, AI enhances the dataset used to train PBPK models, creating more robust models with diverse patient characteristics.

Machine learning techniques further contribute to model optimization and personalization. These techniques enhance prediction accuracy by optimizing PBPK model parameters based on available data. Personalized models consider individual variability factors such as maternal age and genetics, resulting in tailored and precise predictions. Leveraging pre-trained models through transfer learning accelerates model development and improves its relevance to pregnant populations. Deep learning enables the predictive modeling of drug–placenta interactions by analyzing molecular structures, transporter expression, and binding affinities. This capability enhances our understanding of drug transfer across the placenta, crucial for predicting fetal exposure during pregnancy. The integration of real-world data (RWD) through machine learning processes also enriches PBPK models with insights from electronic health records and claims data, providing valuable information on drug exposure, outcomes, and safety during pregnancy. Additionally, AI techniques contribute to model validation and uncertainty quantification by assessing model reliability and identifying areas for improvement. These tools address data limitations, personalize predictions, optimize model performance, and empower informed decision-making regarding treatment efficacy in pregnant individuals [219].

Research has explored the use of machine learning in studying antiviral efficacy in pregnant women. Huang et al. emphasized the need for further research on the use of antiviral drugs during pregnancy, particularly in understanding their pharmacokinetics, safety, and transplacental permeability [220]. Herbek et al. proposed the use of human maternal–fetal interface cellular models to assess the toxicity of antiviral drugs during pregnancy, which could be a potential application of machine learning [221,222]. Mudra et al. discussed the use of antiviral therapy for chronic viral hepatitis B in pregnant women, highlighting the importance of using category B drugs during pregnancy. Young et al. predicted adverse outcomes in pregnant patients positive for SARS-CoV-2 using a machine learning approach to forecast adverse maternal and perinatal outcomes in pregnant individuals infected with SARS-CoV-2. A dataset comprising 1501 SARS-CoV-2-positive cases in pregnancy was analyzed, incorporating various demographic variables, comorbidities, laboratory markers, respiratory parameters, and COVID-19-related symptoms. ML models were trained to predict maternal or fetal/neonatal death or critical illness, with the Random Forest model demonstrating superior performance. This model achieved an accuracy of 89.0%, correctly identifying high-risk and low-risk patients with notable precision and recall rates. The findings from the study exemplify the potential of ML algorithms in prognosticating adverse outcomes in pregnant patients infected with SARS-CoV-2 [223]. By leveraging a comprehensive dataset encompassing diverse clinical variables, ML models could effectively identify individuals at a heightened risk of severe illness or clinical deterioration. This proactive risk assessment facilitates timely interventions, thereby optimizing maternal and neonatal health outcomes [224]. The model’s ability to predict adverse outcomes highlights the value of integrating a range of features, including patient characteristics, symptoms, clinical signs, and laboratory markers. This approach aids clinicians in making informed decisions about hospital admission, treatment initiation, and triaging, improving patient care and resource allocation. The study also underscores the need for comprehensive data repositories and collaborative research to advance ML-driven prenatal care. Pooling diverse data sources can develop robust ML models for complex clinical challenges, provided the data are high-quality and representative to ensure unbiased, generalizable findings.

Regarding the potential of machine learning algorithms in predicting treatment failure in patients with HCV infection, Park et al. developed and validated these algorithms using data from the HCV-TARGET registry, with the elastic net model showing the best performance. Predictors of treatment failure included male sex, treatment duration <8 weeks, treatment discontinuation due to adverse events, advanced liver disease, low albumin, high bilirubin, and substance use [225]. Four machine learning models were compared with multivariable logistic regression, with all machine learning models outperforming the regression model. The elastic net (EN) model was the most parsimonious, requiring the fewest predictors and showing good discrimination performance. Predictors of treatment failure included male sex, treatment duration <8 weeks, treatment discontinuation due to adverse events, advanced liver disease, low albumin, high bilirubin, and substance use. Machine learning models offer a promising approach to identifying patients at risk of DAA treatment failure, informing treatment strategies, and potentially reducing the burden of retreatment in hepatitis C infection [225]. Similarly, Osinubi et al. and Nabulsi et al. also identified predictors of treatment failure, with Osinubi focusing on the use of serial HCV RNA tests and Nabulsi on patient-level variables [226,227]. Harabor et al. extended this work to the prediction of hepatitis B and C seropositivity, demonstrating the potential of machine learning models in this area [228].

Another important consideration is causal inference, which is paramount in pharmacometrics. It focuses on understanding the effects of real and hypothetical interventions concerning drug therapy, underpins discussions on cause-and-effect relationships, confounding factors, and treatment effects, and aids in informed decision-making. Three key concepts in causal inference for pharmacometricians include potential outcomes, the g-formula, and Directed Acyclic Graphs (DAGs) [229]. This is crucial for optimizing drug therapy, especially concerning infections during pregnancy, where the drug exposure’s impact on maternal and fetal health is pivotal. By employing causal models, we can predict drug concentrations, efficacy, and safety during pregnancy, addressing confounding factors. It helps distinguish the actual effects of the drug from other variables that might influence outcomes, ensuring that the observed effects are due to the drug itself. This is particularly relevant as pregnant women have historically been excluded from clinical studies, rendering them “clinical orphans”. Understanding the causal effects of drugs allows for the adjustment of dosing regimens to achieve the desired therapeutic outcomes while minimizing risks. Moreover, causal inference provides a robust framework for predicting how different patient subgroups will respond to treatments, which aids in personalized clinical decision-making and improves overall care. It handles nonrandomized comparisons common in pharmacometrics analyses, enabling regulatory decision-making through techniques like propensity-based matching. By fostering counterfactual thinking, causal frameworks encourage the exploration of alternative scenarios, enhancing our understanding of drug therapy outcomes [230]. Causal machine learning offers flexible, data-driven methods for predicting treatment outcomes, including efficacy and toxicity. It estimates individualized treatment effects, facilitating personalized clinical decision-making based on patient profiles and outcomes, particularly important in estimating treatment effects at various levels and identifying patient subgroups that benefit the most from treatment [231,232]. In the field of pharmacokinetics and drug safety during pregnancy, the development of PBPK models has emerged as a crucial tool. Refer to Figure 3 and Table 3 for key approaches instrumental in developing PBPK models, predicting fetal exposure, and overcoming data limitations in placenta research.

## 6. Future Considerations

Significant strides have been made in understanding antiviral therapy for pregnant women, but further efforts are needed to combat future pandemics and control viral infections by 2030. Ongoing research aims to address ethical drug experimentation and the pharmacokinetics, metabolism, and pharmacological effects of pregnancy to improve care and save lives during outbreaks. To accelerate progress, preclinical studies should be completed earlier, and under certain conditions, pregnant women could be included in phase III trials to obtain crucial safety and efficacy data sooner. Traditionally excluded from clinical trials, pregnant women need to be included with appropriate safeguards to close the data gap on drug safety and efficacy. There is a pressing need for accurate models of human pregnancy, considering the combined effects of pregnancy and other health conditions on physiological changes. Integrating viral infection dynamics into modeling efforts, such as PBPK modeling, shows promise for future research. Our research group is promoting this approach [208,209,233,234,235,236,237,238,239,240]. By refining these models with more data and incorporating viral dynamics, we can optimize antiviral therapy for pregnant women. Challenges include validating PBPK models and addressing gaps in system models, but opportunities lie in simulating outcomes in clinical studies, establishing registries, and informing individualized dosing decisions. In summary, while there are challenges in integrating viral infection dynamics into PBPK modeling, there are also significant opportunities to enhance our understanding of antiviral therapy for pregnant women. Continued research holds promise for developing tailored therapeutic strategies to meet the unique needs of pregnant individuals and improve treatment outcomes. 

## Figures and Tables

**Table 1:** Viral infection dosing regimen recommendations in pregnant women: safety, efficacy notes, and knowledge gaps.

Viral Infection	Dosing Regimen Recommendation in Pregnant Women	Safety and Efficacy Notes	Knowledge Gaps
HIV	Antiretroviral therapy (ART) as per guidelines. Adjustments may be needed based on individual patient factors.	ART generally safe and effective in pregnancy, reducing mother-to-child transmission risk.	Long-term effects of ART on fetus, optimal timing of initiation during pregnancy, impact on maternal health outcomes.
Hepatitis	Vaccination recommended for Hepatitis B. Treatment for Hepatitis C depends on the genotype and stage of liver disease.	Hepatitis B vaccination safe during pregnancy. Limited data on efficacy of Hepatitis C treatment in pregnant women.	Safety and efficacy of Hepatitis C treatment during pregnancy, impact of maternal treatment on vertical transmission.
Influenza	Annual influenza vaccination recommended during pregnancy.	Influenza vaccination safe and effective in pregnant women, reduces the risk of influenza-related complications.	Long-term effects of influenza vaccination on the fetus, optimal timing of vaccination during pregnancy.
CMV	No specific antiviral treatment for CMV during pregnancy.	CMV infection in pregnancy can lead to congenital CMV, causing developmental issues in newborns. Prevention through hygiene measures advised.	Development of safe and effective antiviral therapy for CMV during pregnancy, understanding of immune responses to prevent congenital CMV transmission.
SARS-CoV-2	Vaccination recommended during pregnancy. Treatment varies based on severity and trimester.	Limited data on SARS-CoV-2 vaccines during pregnancy; preliminary studies suggest safety and efficacy. SARS-CoV-2 infection in pregnancy associated with increased risk of complications.	Long-term effects of SARS-CoV-2 vaccination on pregnancy outcomes and fetal development, optimal management strategies for SARS-CoV-2 in pregnant women.

**Table 2:** Overview of prophylactic and therapeutic vaccines for viral infections during pregnancy.

Viral Infection	Prophylactic Vaccines	Therapeutic Vaccines
HIV	No prophylactic vaccine is available. However, it would be crucial to prevent vertical transmission from mother to child during pregnancy and childbirth.	No therapeutic vaccine is available. May have a role in managing maternal infection and reducing the risk of vertical transmission (research is still ongoing).
HBV/HCV	Recommended for pregnant women at high risk of exposure to prevent transmission to the fetus during pregnancy and childbirth.	No therapeutic vaccine is available. It can have a limited role, as the focus is on managing the infection through antiviral therapy.
Influenza	It is recommended that all pregnant women protect themselves and their newborns from severe influenza complications.	No therapeutic vaccine is available. May have a limited role, as the focus is on preventing severe disease through prophylactic vaccination.
CMV	No prophylactic vaccine is available. But it would be crucial to prevent congenital CMV infection, a leading cause of birth defects.	No therapeutic vaccine is available. May have a role in managing maternal infection and reducing the risk of vertical transmission.
SARS-CoV-2	Strongly recommended for pregnant women protect them and their newborns from severe SARS-CoV-2 disease.	No therapeutic vaccine is available. May have a role in managing acute SARS-CoV-2 infection in pregnant women, but more research is needed.
RSV	Prevent RSV infection in infants by providing passive immunity through maternal vaccination during pregnancy, reducing the risk of infection in infants.	No therapeutic vaccine is available. May have a limited role, as the focus is on preventing RSV infections in infants in their vulnerable early months.

**Table 3:** Challenges in utilizing PBPK models to predict treatment efficacy in viral infections during pregnancy.

Limitation	Description
Lack of data	Delay in data availability due to pregnant women’s exclusion from clinical trials; hampers accurate modeling of drug pharmacokinetics.
Insufficient characterization of physiological changes	Inadequate characterization of pregnancy-related physiological changes like plasma volume and protein concentrations, impacting model accuracy.
Unavailability of gestational-age dependent equations	Lack of equations for enzymes like CYP2C19, CYP2B6, and CYP2C9 at different gestational stages, limiting model predictability.
Limited availability of clinical data	Sparse clinical PK data, particularly for drugs like sertraline, hindering model performance assessment.
Uncertainty in hepatic intrinsic clearance	Variability in hepatic intrinsic clearance parameter estimation affecting model precision; integration of additional CYP data can enhance accuracy.
Inadequate representation of placental transfer	Simplified models and scarce data on placental transfer parameters limit fetal exposure prediction accuracy.
Uncertainty in maternal and fetal pharmacokinetics	Uncertain maternal and fetal pharmacokinetics influencing drug exposure prediction; integration with placenta perfusion data improves accuracy.
Lack of published PBPK models for mabs in pregnancy	The absence of PBPK models for monoclonal antibodies (mAbs) in pregnant women highlights a knowledge gap and modeling challenge.
Need for suitable modelling and simulation techniques	Requirement for suitable techniques to optimize PBPK models predicting mAb exposure in pregnant women; ongoing research needed.
Challenges in incorporating pharmacodynamic changes	Impact of pregnancy-related pharmacodynamic changes on drug response; PBPK models need to consider these alterations.
Limitations in extrapolating data from healthy pregnant women	Data limitations from healthy pregnant women with singleton births; maternal health complexities not fully accounted for.
Need for incorporating placental transfer into PBPK models	Crucial for simulating fetal exposure; ex vivo human cotyledon perfusion models provide valuable data.
